# Preoperative cardiopulmonary exercise testing in England – a national survey

**DOI:** 10.1186/2047-0525-2-4

**Published:** 2013-02-25

**Authors:** Sam Huddart, Emily L Young, Rebecca-Lea Smith, Peter JE Holt, Pradeep K Prabhu

**Affiliations:** 1 Department of Anaesthesia, Royal Surrey County Hospital, Guildford, GU2 7XX, UK; 2 Department of Anaesthesia, Mount Sinai Hospital, Toronto, Canada; 3 St George’s Vascular Institute, London, UK; 4 The Queen Elizabeth Hospital, Adelaide, Australia

**Keywords:** Cardiopulmonary exercise testing, Exercise testing, Preoperative assessment, Preoperative risk assessment, National survey

## Abstract

**Background:**

Cardiopulmonary exercise testing (CPET) has become well established in the preoperative assessment of patients presenting for major surgery in the United Kingdom. There is evidence supporting its use in risk-stratifying patients prior to major high-risk surgical procedures.

We set out to establish how CPET services in England have developed since the only survey on this subject was undertaken in 2008 (*J Intensive Care Soc* 2009, 10:275–278).

**Methods:**

Availability of preoperative CPET and contact details were collected via a telephone survey and email invites to complete the online survey were sent to all contacts. The survey was live during March and April 2011.

**Results:**

We received 123 (74%) responses from the 166 emails that were sent out. In total, 32% (53/166) of all adult anesthetic departments in England have access to preoperative CPET services and a further 4% (6) were in the process of setting up services. The number of departments offering preoperative CPET, including those in the process of setting up services, has risen from 42 in 2008 to 59 in 2011, a rise of over 40%. Only 61% of the clinics are run by anesthetists and 39% of clinics have trained cardiorespiratory technicians assisting in the performance of the test. Most of the clinics (55%) rely solely on a bicycle ergometer. Vascular surgical patients are the largest group of patients tested, and the majority of tests are run to a symptom-limited maximum. We estimate that 15,000 tests are performed annually for preoperative assessment in England. Only 37% of respondents were confident that the tests performed were being billed for.

**Conclusions:**

CPET is increasing in popularity as a preoperative risk assessment tool. There remains a lack of consistency in the way tests are reported and utilized. The results highlight the extent and diversity of the use of preoperative CPET and the potential for further research into its use in unstudied patient groups.

## Background

Cardiopulmonary exercise testing (CPET) has become well established in the preoperative assessment of patients presenting for major surgery in the United Kingdom. There is some evidence to support the use of CPET-derived variables for risk stratification and allocation to an appropriate level of post-operative care in many major surgical specialties [1-18], although there are no published randomized control trials. To date there has been one published survey into the use of preoperative cardiopulmonary exercise testing (PCPET) in England (performed in 2008, published in 2009) [19]. This survey identified 30 units in England that provided PCPET services for preoperative assessment, and a further 12 units in the process of setting up a service. We set out to determine how PCPET services have progressed since the 2008 survey and to ascertain the wider interest in PCPET in England.

## Methods

The survey was conducted in two main stages. The first stage involved telephoning anesthetic secretaries in every NHS trust in England. This way, we hoped to find out how many departments performed PCPET, and to obtain contact details for a named consultant responsible for the service. The second stage involved sending a link to an online survey to each of these contacts. The National Research Ethics Service (NRES) has confirmed that, in accordance with their guidelines, the survey does not require formal ethical approval.

### Telephone survey

We accessed the NHS England website in December 2010 [20]. This website lists details of all 168 NHS trusts in England, 159 of which provide surgical services for adult patients. We contacted these trusts by telephone and identified 166 functionally separate anesthesia departments providing services for surgical procedures in adults. We contacted the anesthetic secretaries for all of these departments by telephone asking if preoperative CPET is available in their department and for contact details of the clinician responsible for either CPET or preoperative assessment.

### Online survey

We composed the survey using an online survey tool (http://www.surveymonkey.com). For the majority of questions, we used a multiple-choice format with an additional free-text option for comments. Not all questions were compulsory for submission of the completed survey. The survey questions and structure are shown in Additional files 1 and 2.

The survey was sent to each identified email contact as a link within the email. A maximum of three reminder emails were sent over the following four weeks. The survey was live in March and April 2011.

### Repeat telephone survey

Some of the secretaries contacted in the original telephone survey did not know if their department had access to PCPET. If we did not receive a response to the online survey and were originally unsure if they had PCPET services, we re-contacted them by telephone, so that we could confirm the total number of departments in England with PCPET services.

## Results

We contacted all 166 (100%) anesthesia departments by telephone. In total (after the repeat telephone survey described above) we identified 53 departments who offer PCPET (32%).

### Online survey

We received a total of 128 responses to the survey. Five of these were duplicate responses from individuals in the same department. Only the first response received from each of these departments was included in the analysis. As such our overall response rate was 74% (123/166).

We received 49 (40% of total) responses from departments that offer PCPET and 74 (60% of total) responses from departments without access to PCPET services.

### Departments without PCPET services (*n* = 74)

Thirty-five (47%) of those who responded have made an attempt to set up PCPET services that was unsuccessful. The reasons given for failed attempts included: financial (43%), perceived lack of clinical need (11%) and insufficient evidence of benefit (6%).

Thirty-three departments (45%) have not attempted to set up a service. Reasons stated for this included: financial constraints or lack of resources (39%), inappropriate case mix (9%), training issues (3%), lack of support from other departments (3%) and conflicting evidence for clinical benefit (3%).

Six departments (8%) are in the process of setting up a peri-operative CPET service.

**Figure 1:**
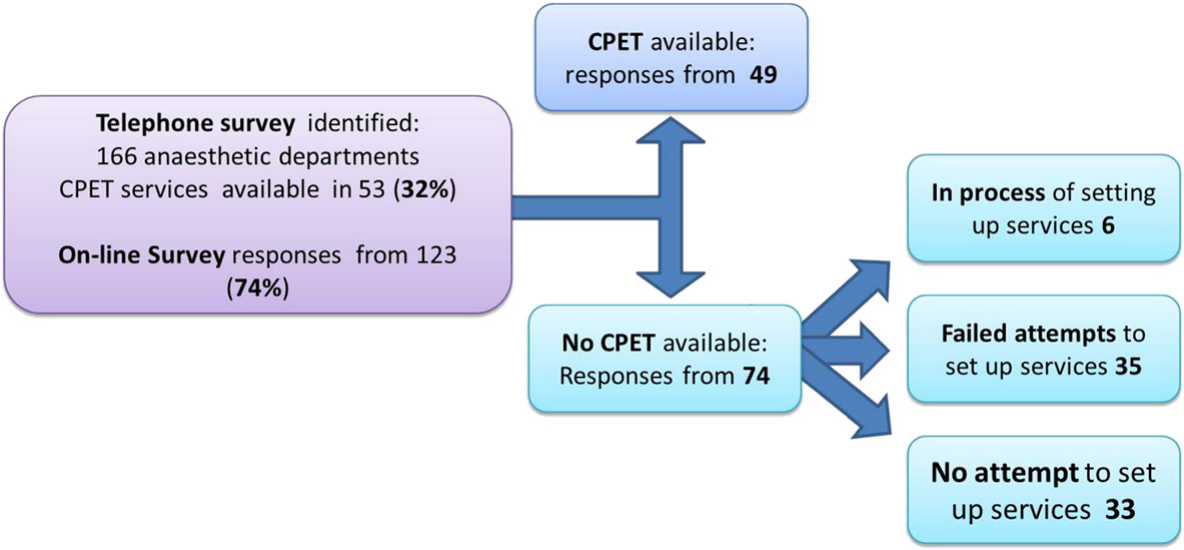
Survey response rates.

The survey responses rates are summarized in Figure 1.

### Departments with PCPET services (*n* = 49)

The majority of respondents to the survey were anesthetists (90%), the remainder being clinical scientists (6%) and physicians (4%).

#### Logistical aspects of PCPET services

Forty-five respondents (92%) indicated that testing is performed in-house. One department refers patients to a private CPET clinic as well as testing patients in-house themselves.

**Figure 2:**
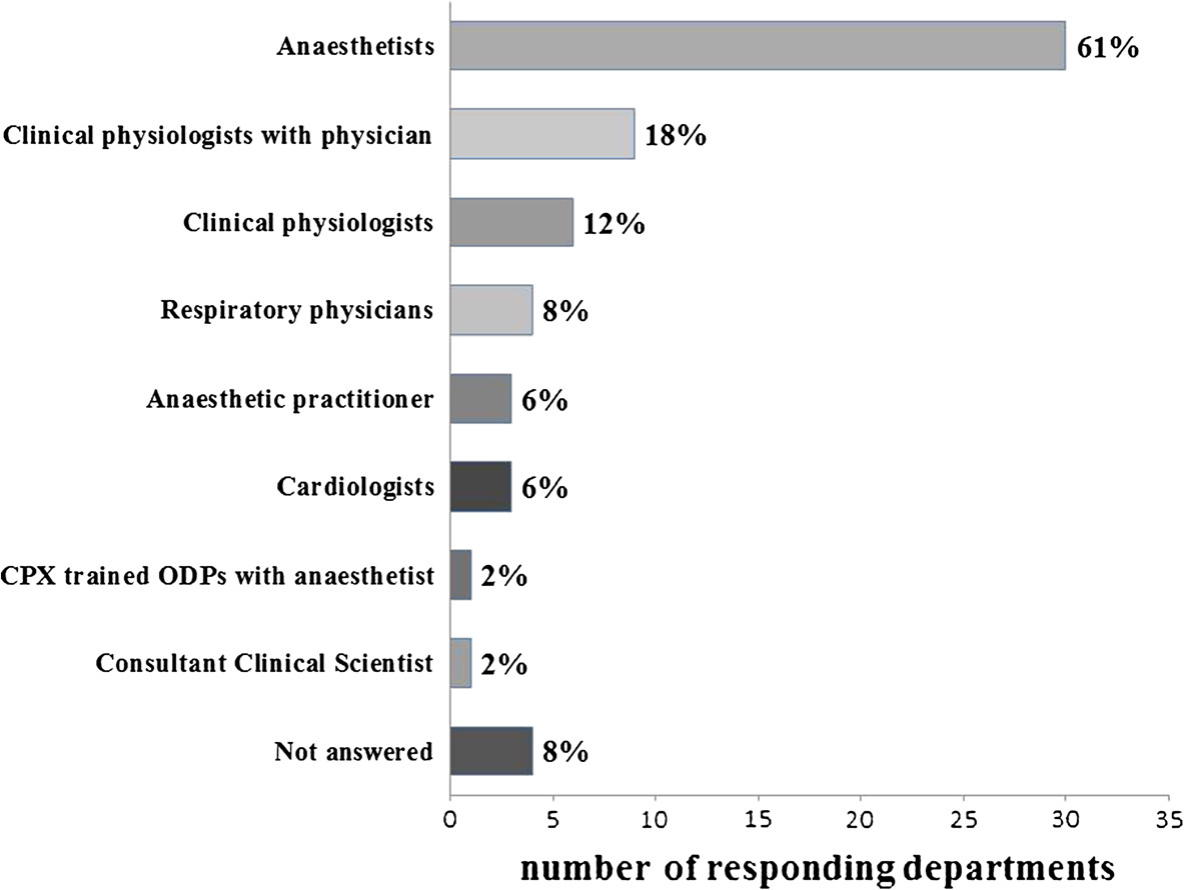
Who performs preoperative cardiopulmonary exercise testing?

The majority of tests are conducted by anesthetists. Some are conducted by a variety of other clinicians and non-clinicians (Figure 2). Three respondents do not have any assistance during testing (6%) and 19 (39%) are assisted by a trained cardio-respiratory technician. Other assistance during testing includes: operating department practitioners (14%), pre-assessment nurse (14%), nursing auxiliary (6%), anesthetic practitioner (2%), physicians’ assistants in anesthesia (2%) and research nursing staff (2%).

Clinics are operated in a variety of locations: in the pre-assessment clinic (39%), in the respiratory laboratory/clinic (27%), in the cardiology department (12%), in a ward area (4%), in the outpatients department (4%), in a research laboratory (2%) and on the intensive care unit (2%). The responsibility for maintenance, cleaning and sterilization of reusables and stocking of disposables lies with the clinician (20%), technician (49%) or a nurse (10%).

#### Clinical aspects of PCPET services

Referrals for CPET are received from multiple sources. Departments receive referrals from surgical colleagues (76%), anesthetic colleagues (69%), the pre-assessment clinic (67%) and from multi-disciplinary team meetings (35%). Interestingly only 22% of departments include strict predetermined criteria as a part of their referral pathway. Of the total, 8% reported receiving referrals from cardiologists and respiratory physicians.

Twenty-four (49%) departments consent their patients verbally for PCPET, 11 (22%) require formal written consent and seven (14%) do not consent their patients prior to testing. This is a significant deviation from the practice in most cardiology exercise labs, where formal consent is not obtained prior to a treadmill test.

All respondents have access to a cycle ergometer for testing. However, the majority (55%) of departments only have access to a cycle ergometer, thereby limiting the range of patients who are physically able to perform the test. In addition to a cycle ergometer, eight (16%) departments have access to a hand crank ergometer and seven (14%) have access to a treadmill ergometer. One department (2%) has access to bicycle, hand crank and treadmill ergometers.

**Figure 3:**
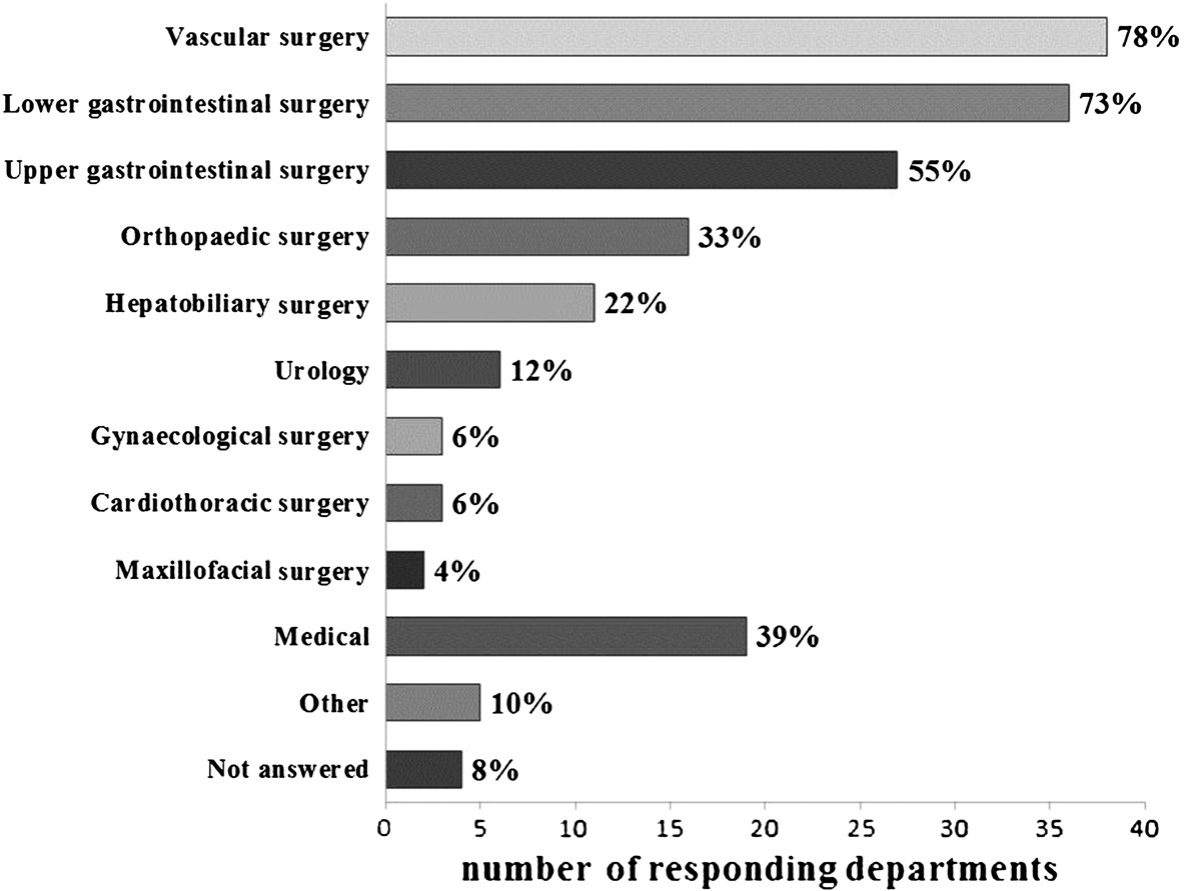
Which subspecialty groups are tested with PCPET?

A variety of sub-specialty patient groups are tested, as depicted in Figure 3. Other patient groups tested are pediatric cardiology, ICU follow-up and adult congenital heart disease follow-up (Figure 3).

The majority of respondents use the anaerobic threshold (AT) (90%), Ve/VCO2 (71%), peak VO2 (59%), and Ve/VO2 (31%) to risk-stratify patients. A variety of other CPET-derived parameters were also reported as being used for risk stratification. For example: onset of ischemia, oxygen pulse, heart rate response, blood pressure response, desaturation, ventilator limitation, VO2/work rate slope and Ve/VCO2 slope.

The majority of respondents run their tests to the patients’ symptom limited maximum (71%). This provides evidence for the need for a clinician to be present during the test on the grounds of safety. Some terminate tests after the patient has exercised to their anaerobic threshold (14%) or to their target peak VO2 (6%). Other responses include achieving predicted maximal heart rate and ischemic ECG changes.

The majority of respondents use the results for individual patient risk stratification and counseling (86%) and to allocate patients to an appropriate level of post-operative care (84%). Some departments use the results to determine the level of intraoperative monitoring (47%). Other uses reported by respondents include: for clinical diagnostics, to modify surgical procedure (in support of funding applications for less invasive procedures in high risk cases) and to assess the need for pre-optimization.

The majority do not recommend cancellation of an individual case based on the CPET result (55%). However, 33% of respondents do recommend cancellation of cases based on individual CPET results. There were many comments left for this question implying that the decision to cancel cases is more complex than this. The comments highlighted that CPET results advise risk and that the final decision lies with the patient, surgeon and, in some cases, the anesthetist. Some respondents felt strongly that the decision to cancel a case is not for the CPET clinic.

**Figure 4:**
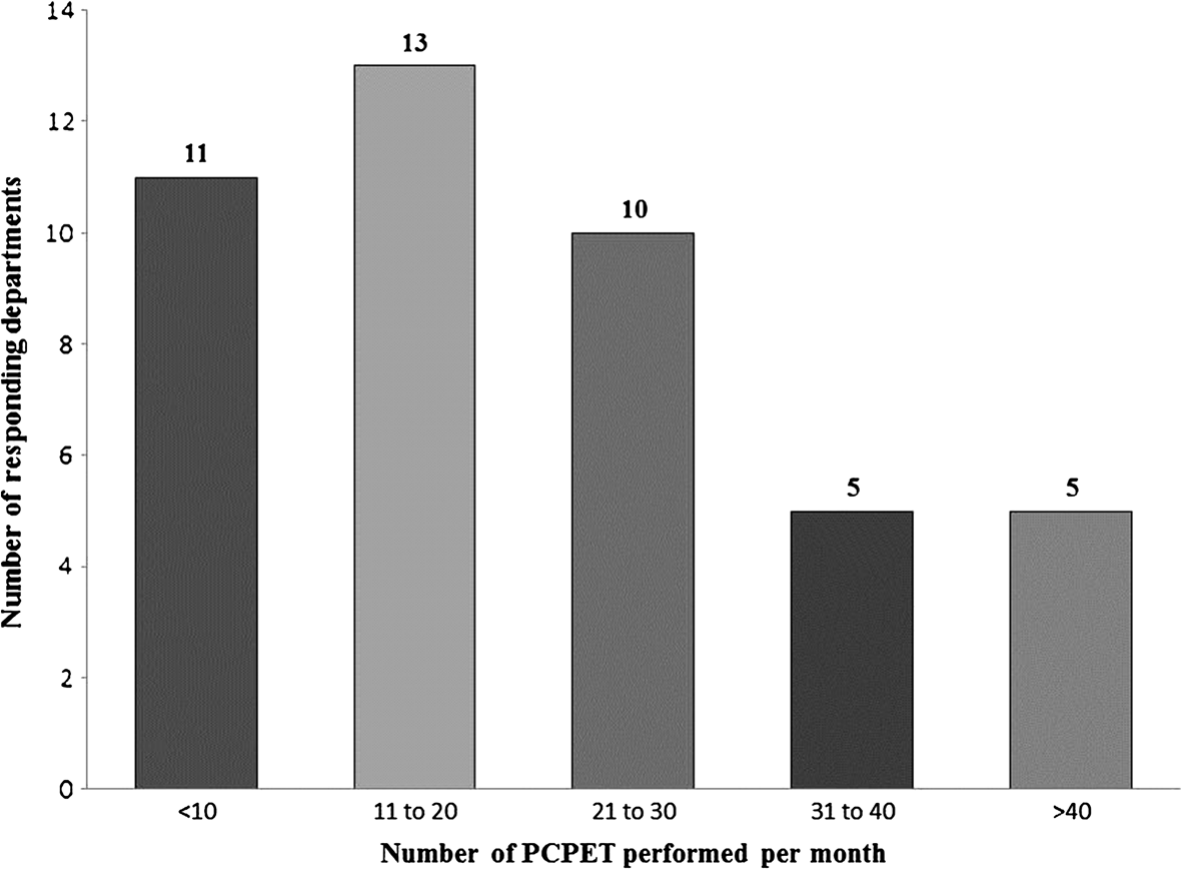
Peri-operative cardiopulmonary exercise testing output per month in England.

The median number of tests performed per clinic session was 3 (range 0.5-7.5, mean 3.2, mode 3, SD 7.3). The median number of PCPET clinic sessions per month was 6 (range 0.5-40, mean 8.4, mode 4, SD 1.5). Of the 53 departments identified by the telephone survey we received 49 responses. Of these 42 gave estimates regarding the output of testing services. If we substitute mode values (most conservative estimate) for missing data we estimate that over 15,000 PCETs are performed each year in England (Figure 4).

#### Administrative and managerial aspects of PCPET services

We were keen to find out how the administrative aspects of these clinics were managed. The questions ranged from organizing appointments to calibrating and validating the equipment and getting the reports to the referring clinician.

Appointments are arranged using written, formal appointment letters with an information leaflet in the majority of cases (76%). Other methods include: by telephone (8%) or written, formal appointment letters without information leaflet (8%).

The PCPET equipment is owned either by anesthetic departments (51%), respiratory departments (20%), cardiology departments (10%), clinical measurement/physiology departments (4%), surgical departments (2%) or clinical research facilities (2%).

Respondents reported a variety of administrative support as part of their service infrastructure. These include: departmental secretary (39%), pre-assessment clinic staff (27%) and own secretary (10%). Several respondents commented that administrative tasks are undertaken by clinicians. Nine services (18%) have no administrative support.

Where anesthetists perform PCPET, the majority of sessions are considered as a clinical professional activity (51%) in job plans. Some sessions are in supporting professional activity time (12%), and others in the clinicians’ own time (8%).

The majority of tests performed are logged onto the hospitals’ patient administration systems (67%); 12% are not logged and 10% of respondents did not know if tests were logged.

The primary care trust (PCT) is billed for the patients tested in only 37% of departments. No payment is received for testing in 31% of responding departments and 24% of respondents did not know if the PCT was billed.

## Discussion and conclusion

We contacted 100% of individual anesthetic departments and identified 32% with CPET services and 4% in the process of setting up. In the 2008 survey Simpson *et al*. contacted 89% of trusts in England by telephone and received a 66% response rate when asking about the availability of CPET [1]. The higher response rate in our survey could be attributed to persistence with the telephone survey, the inclusion of a repeat telephone survey and accessibility of the online survey. The number of departments offering CPET has increased from 17% in 2008 to 32% in 2011 (while those in the process of setting up increased from 23% in 2008 to 36% in 2011). However, the 2008 survey may well have underestimated the availability of CPET due to their lower response rate. It is also unknown if any departments in the process of setting up a service were indeed successful. The 2008 survey was structured as nine open questions and sent as an email attachment. The authors commented that some responses were brief and lacked detail. In contrast our survey was online and included mostly multiple-choice questions with the opportunity for comment where appropriate. We believe this method increases the consistency of responses. However, we acknowledge that this style of questioning can be leading and may not collect some details of uncommon practice. The 2008 survey identified specific thresholds for parameters used for risk stratification, our survey did not. We have shown that the majority of patients tested have vascular, lower gastrointestinal or upper gastrointestinal symptoms. It appears that there is an extrapolation of the evidence to other unstudied surgical groups. The published evidence shows that different CPET parameters have variable predictive power in different sub-specialties [2-18]. Therefore, we believe extrapolating specific evidence to unstudied patient groups is not advisable.

We were surprised to find that the responses from departments without PCPET suggest that there is a desire to have these services in the majority of departments, but that the primary obstacles are financial. Some departments may not have the high-risk case load to justify their own service, although this was not commented on by any respondents. The results suggest that there is an interest in PCPET in the majority of anesthetic departments, but that the perceived obstacles are the financial implications of setting up such a service. However, the survey was targeted at those with an interest in preoperative assessment. This group may be more enthusiastic and not represent the views of all anesthetists.

Not all questions in the survey were compulsory. While this ensured a better response rate overall, we did receive responses with some unanswered questions. The median number of unanswered responses per question was four (8%) (range 4–9, mean 4.9, SD 1.2) and this accounts for the shortfall in percentages of responses given in the results section.

There are a number of patient groups in whom PCPET does not have published outcome correlation data. These include patients requiring hepatobiliary, maxillofacial, gynecological or urological surgery. This highlights the importance of and potential for, further research into PCPET and post-operative outcomes, particularly in unstudied groups.

Departments use a wide variety of PCPET parameters to risk-stratify individual patients. Some parameters used are well established in the literature as good predictors of outcome (for example AT and Ve/VCO2) [2-11,14-18]. However, some studies suggest that AT is not a consistently strong predictor across all surgical groups [12,13]. Other parameters are also used to predict risk without published evidence, for example oxygen pulse. The variability of parameters used by respondents seems to reveal confusion over how results should be used to guide management. This is reflected in the inconsistencies of the reported predictive power of individual parameters published in the literature [2-18]. It would be interesting to know how many respondents use combinations of parameters or individual parameters alone (e.g. AT, Ve/VCO_2_, peak VO_2_ or Ve/VO_2_) to risk-stratify patients.

Results are used for individual patient risk stratification and to allocate patients to the appropriate post-operative level of care. Some departments reported using results to determine the level of intra-operative monitoring. We are not aware of any specific evidence supporting this practice. One department reported using the results to support applications for funding for less invasive surgery (e.g. endovascular aortic aneurysm repair) in cases identified as high-risk cases by PCPET; again we are not aware of any evidence supporting this.

There appears to be controversy regarding the recommendation to cancel patients based on PCPET results. The majority of departments (55%) do not recommend cancellation of individual cases solely on the basis of PCPET results. A third of departments do recommend cancellation on the basis of PCPET results. The comments suggest that those who cancel patients on the basis of test results do so in conjunction with surgical colleagues and other clinical patient information available to them.

We were surprised to see the estimated number of tests performed in England per year is in excess of 15,000. Since we used modal values for missing data it is likely to be a conservative estimate. The numbers given in the survey responses do not necessarily take into account seasonal variations in output (e.g. bank holidays, annual leave and cancellations) and therefore may be overestimates. Even if we assume a month of testing is lost to these factors, the estimate is still in excess of 14,000 tests performed per year in England.

The number of tests, and the breadth of specialty groups in which testing is used, represents an exciting opportunity for collaborative research. Recent national attempts to establish communication and data sharing between departments will be a welcome step towards this goal (National PCPET Meeting, July 2011).

We were surprised to see that not all services are appropriately remunerated for the tests performed. Of respondents, 31% reported receiving no funding for tests from the PCT. Indeed a further 24% of respondents did not know if payment for tests was received from the PCT. We perceive this as being one of the key messages from the survey. Given that a significant proportion of respondents have indicated financial constraints as being the major hurdle in setting up these services, we were surprised to find that more than half the existing services were not generating income from their tests.

The survey has demonstrated the rapid growth of PCPET as a preoperative risk assessment tool in England. It has identified financial constraints as the main obstacle in setting up new services. This is despite clear evidence that there is significant national interest in having PCPET services available for the preoperative risk assessment of high-risk patients. We hope the results of the survey will add to the evidence presented in support of future attempts to establish new services.

## Abbreviations

AT: anaerobic threshold; CPET: cardiopulmonary exercise testing; ECG: electrocardiogram; NHS: National Health Service; PCPET: preoperative cardiopulmonary exercise testing; PCT: primary care trust; Ve/VCO2: ventilatory equivalents for carbon dioxide; Ve/VO2: ventilatory equivalents for oxygen; VO2: oxygen consumption (liters per minute).

## Competing interests

PJEH is on the editorial board of the *Perioperative Medicine Journal*.

## Authors’ contributions

SH co-wrote and designed the survey (with PKP), collected contact details and data, coordinated the data collection, interpreted data and drafted the manuscript. ELY collected contact details and data, independently validated data interpretation and helped in the draft of the manuscript. RS collected contact details and data, independently validated data interpretation and helped in the draft of the manuscript. PJEH independently validated data interpretation and helped in the draft of the manuscript. PKP conceived of the study, co-wrote and designed the survey with SH, coordinated data collection, independently validated data interpretation, drafted the manuscript and supervised the project. All authors read and approved the final manuscript.

## Authors’ information

SH, RS and ELY are anesthetic trainees at the St Georges School of Anaesthesia, London, UK. PH is an NIHR Clinical Scientist at the St George’s Vascular institute and is currently working at the Queen Elizabeth Hospital, Adelaide, Australia. PKP is a consultant anesthetist and cardiopulmonary exercise testing lead at the Royal Surrey County Hospital, Guildford, Surrey, UK.

The results of this survey were presented by the authors as a poster and an oral presentation at the 4th National CPET Forum, the Institute of Education, London on 6 July 2011.

## Supplementary Material

Additional file 1 **CPET survey flow chart.** Additional file 1 - CPET survey flowchart.docx. Click here for file

Additional file 2 **Cardiopulmonary exercise testing survey questions and structure.** Additional file 2 - CPET survey structure and content.docx. This additional content details the survey structure and content. It contains a flow chart of the survey structure and a list of the survey questions and answer options. This report is independent research supported by the National Institute for Health Research (NIHR) Clinical Scientist – (NIHR CS-011-008). The views expressed in this publication are those of the author(s) and not necessarily those of the NHS, the National Institute for Health Research or the Department of Health. Click here for file
