# Supplementary figures and images for: Preoperative cardiopulmonary exercise testing in England – a national survey
Source: Perioper Med (Lond). 2013 Feb 25;2:4. doi: 10.1186/2047-0525-2-4 (PMC3964325; doi:10.1186/2047-0525-2-4)

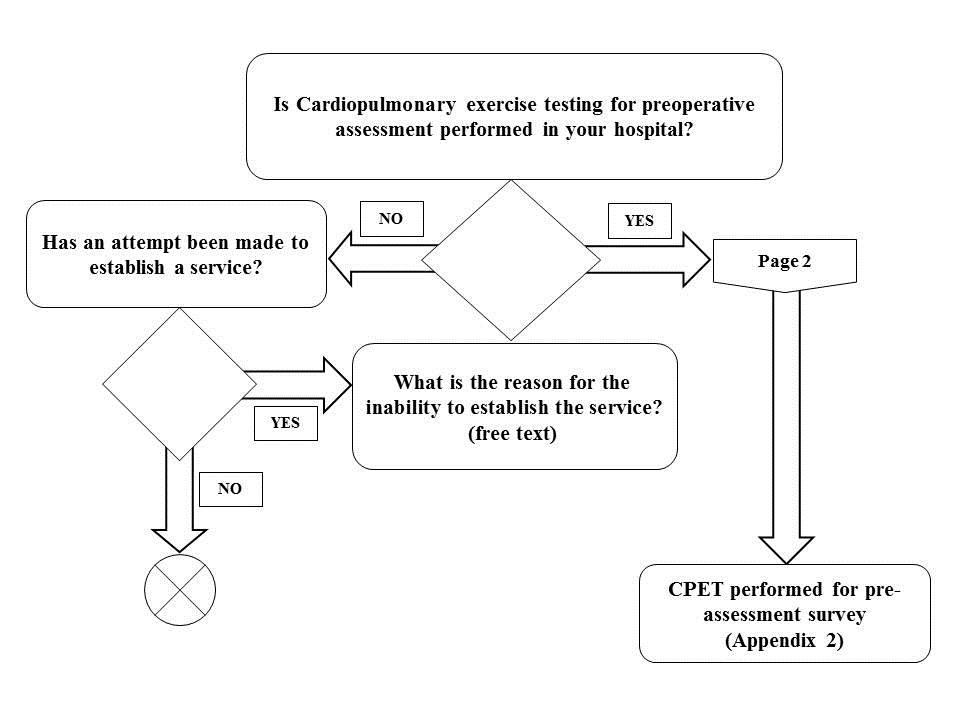

Supplement: Additional file 1 — CPET survey flow chart. Additional file 1 - CPET survey flowchart.docx. [file 2047-0525-2-4-S1.jpeg]
