# Supplementary material for: Preoperative cardiopulmonary exercise testing in England – a national survey
Source: Perioper Med (Lond). 2013 Feb 25;2:4. doi: 10.1186/2047-0525-2-4 (PMC3964325; doi:10.1186/2047-0525-2-4)
Supplement: Additional file 2 — Cardiopulmonary exercise testing survey questions and structure. Additional file 2 - CPET survey structure and content.docx. This additional content details the survey structure and content. It contains a flow chart of the survey structure and a list of the survey questions and answer options. This report is independent research supported by the National Institute for Health Research (NIHR) Clinical Scientist – (NIHR CS-011-008). The views expressed in this publication are those of the author(s) and not necessarily those of the NHS, the National Institute for Health Research or the Department of Health. [file 2047-0525-2-4-S2.docx]

**Additional file 2:**

**Cardiopulmonary Exercise Testing survey questions and structure**

1. Is it performed:

- In house in your own hospital
- Referred to a neighbouring NHS centre
- Referred to a private CPET clinic

2. Is it performed by:

- Anaesthetists
- Respiratory physicians
- Cardiologists
- Clinical physiologists with physician in attendance
- Clinical physiologists without physician in attendance
- Others (please specify)

3. Who owns the machine?

- Anaesthetists
- Respiratory Physicians
- Cardiologists
- Others/Don’t know

4. How do you take referrals? (more than one if appropriate)

- From the preassessment clinic (PAC)
- From anaesthetic colleagues
- Based on strict, predetermined criteria
- From surgeons
- From MDTs
- From adjacent hospitals
- Others (please specify)

5. How many clinics/month are run (on an average, in your hospital)

(free text)

6. How many patients do you test/ clinic (on an average)

(free text)

7. What is the location of your clinic

- PAC
- Respiratory clinic
- Cardiology lab
- Others (eg. Broom Cupboard!!) (please specify)

8. Do you have technician or other help during the clinic

- None
- Trained cardio/respiratory technician
- ODP
- PAC nurse
- Nursing auxillary

9. Who has designated responsibility for maintenance/cleaning/sterilization of re-usables and stocking of disposables?

- Clinician
- Technician
- Nurse
- None

10. Do you have any designated administrative support for taking referrals, sending out appointments, typing out and sending reports etc?

- Own secretary
- Departmental secretary
- PAC secretary
- Others (please specify)
- None

11. How do you make appointments?

- Telephone
- Written, formal appointments with information leaflet
- Written, formal appointments, without information leaflet

12. Ergometers used/available (please indicate all those available)

- Bicycle
- Treadmill
- Hand Crank

13. Do you formally consent your patients prior to the test?

- None
- Verbal
- Formal, written

14. What protocol do you use to decide the point at which to terminate the test?

- Symptom limited maximum
- To just beyond Anaerobic Threshold
- To target Heart Rate
- To target Peak VO2

15. What parameter obtained from the test do you use to risk stratify?

- Anaerobic Threshold
- Peak VO2
- Ve/VCO2
- Ve/VO2
- Others (please specify)

16. In your report, do you recommend cancellation of a case deemed high risk by CPET?

- Yes
- No

(free text)

17. If anaesthetists cover CPET sessions, how are these sessions considered in your job plans:

- SPA
- Clinical PA
- Own time

18. Are the sessions logged on to the hospital’s patient administration system

- Yes
- No
- Don’t Know

19. Is the PCT billed for the patients that are tested in your clinic?

- Yes
- No
- Don’t Know
